# Mitochondrial Targeting in Neurodegeneration: A Heme Perspective

**DOI:** 10.3390/ph11030087

**Published:** 2018-09-18

**Authors:** Veronica Fiorito, Deborah Chiabrando, Emanuela Tolosano

**Affiliations:** Department of Molecular Biotechnology and Health Sciences, Molecular Biotechnology Center, University of Torino, 10126 Torino, Italy; deborah.chiabrando@unito.it (D.C.); emanuela.tolosano@unito.it (E.T.)

**Keywords:** neurodegeneration, mitochondria, therapy, heme, haem

## Abstract

Mitochondrial dysfunction has achieved an increasing interest in the field of neurodegeneration as a pathological hallmark for different disorders. The impact of mitochondria is related to a variety of mechanisms and several of them can co-exist in the same disease. The central role of mitochondria in neurodegenerative disorders has stimulated studies intended to implement therapeutic protocols based on the targeting of the distinct mitochondrial processes. The review summarizes the most relevant mechanisms by which mitochondria contribute to neurodegeneration, encompassing therapeutic approaches. Moreover, a new perspective is proposed based on the heme impact on neurodegeneration. The heme metabolism plays a central role in mitochondrial functions, and several evidences indicate that alterations of the heme metabolism are associated with neurodegenerative disorders. By reporting the body of knowledge on this topic, the review intends to stimulate future studies on the role of heme metabolism in neurodegeneration, envisioning innovative strategies in the struggle against neurodegenerative diseases.

## 1. Implication of Heme in Neurodegeneration

**Figure 1:**
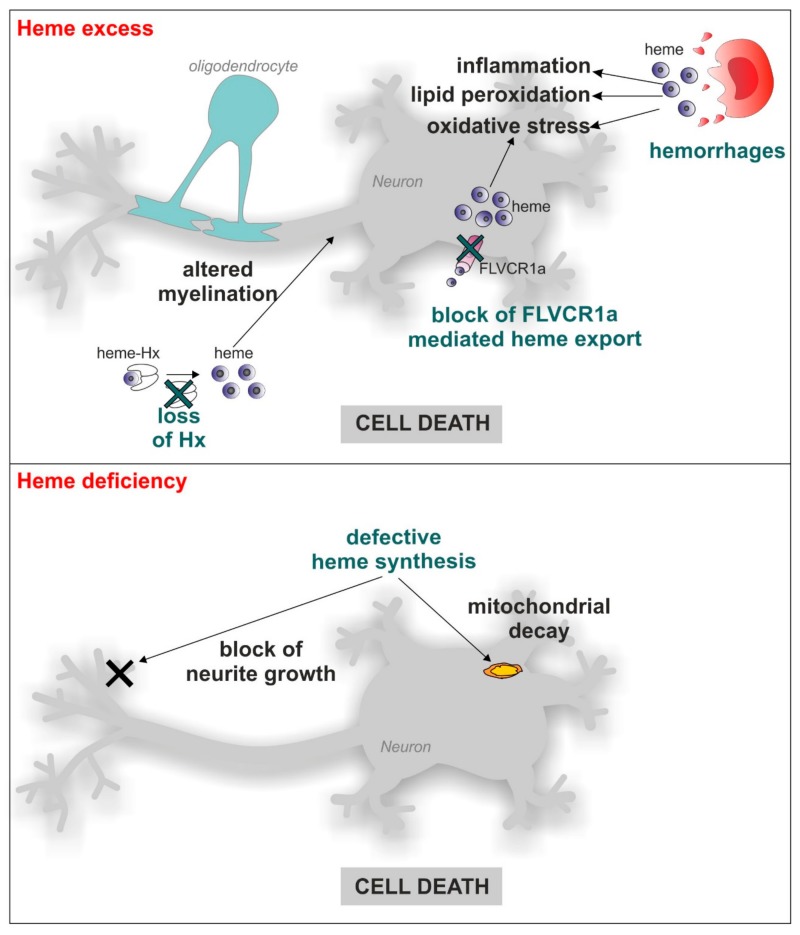
Implication of heme in neurodegeneration. Both heme excess and heme deficiency contribute to neurodegeneration. Heme released during hemorrhages leads to inflammation, lipid peroxidation and oxidative stress; the loss of the heme scavenger Hx causes defective myelination of axons; the impairment of intracellular heme export by FLVCR1a is associated with increased oxidative stress. On the other hand, heme deficiency, due to defective synthesis, leads to mitochondrial decay and the blocking of neurite growth. These events all result in neuronal cell death. In the figure, neurons are represented as the main target for heme-mediated effects; however, other cell types of the nervous system could be affected by the same phenomena.

Heme is a molecule composed by protoporphyrin IX and iron produced by all the cells in the organism, including neurons. Heme mediates a series of functions that encompass oxygen transport, the regulation of gene expression and the modulation of enzyme activity, just to cite the most relevant ones. Moreover, heme can also promote oxidative stress, thus performing as a double-face molecule with both positive and negative properties [1]. This concept is also true for neuronal cells. Indeed, on one hand heme is required for the survival and differentiation of neuronal cells, as demonstrated by the observation that heme deficiency interferes with neurite outgrowth in nerve growth factor (NGF)-induced PC12 cells [2,3] and results in apoptosis in PC12 pheochromocytoma cells, SHSY5Y neuroblastoma cells and U373 astrocytoma cells, as well as in rat primary hippocampal neurons [2,3,4]. However, on the other hand, an excess of free-heme is associated with neurodegeneration. The large amount of hemoglobin and heme released in the brain during intracerebral or subarachnoid hemorrhages promotes oxidative stress, lipid peroxidation, inflammatory response and finally, neuronal cell death [5,6,7]. Moreover, loss of the heme scavenger hemopexin (Hx) causes defective myelination in mice [8,9,10]. Furthermore, impairment of cellular heme export reduces SHSY5Y cells survival [11]. Together, these data indicate that both heme deficiency and excess are deleterious for the survival of neuronal cells (Figure 1), thus suggesting that heme levels must be finely controlled both at the systemic and cellular level. At the systemic level, circulating free-heme is scavenged by the plasma proteins haptoglobin and hemopexin [6,7,8,10,12,13,14]. However, at cellular level, the amount of intracellular free-heme (*labile heme* or *heme regulatory pool*) is regulated at multiple steps: heme synthesis, incorporation into hemoproteins, catabolism, import and export [1,15]. Although this tight regulation has been extensively studied in non-neuronal cells, similar mechanisms likely occur in the nervous system. Indeed, the main proteins involved in the control of labile heme are also expressed in the nervous system [16].

Neurodegenerative disorders are a common and growing cause of mortality and morbidity worldwide [17]. Recently, a series of rare neurodegenerative disorders have been directly linked to alterations of heme metabolism (see Table 1). Defective heme synthesis causes porphyrias, some of which are associated with a wide array of neurological disturbances involving both the central and peripheral nervous systems (neuropathic porphyria). Neuropathic porphyria includes acute intermittent porphyria (AIP), hereditary coproporphyria (HCP), variegate porphyria (VP) and 5-aminolevulinate dehydratase deficiency (ALAD deficiency) [18,19,20]. Furthermore, reduced heme synthesis has been observed in Friederich Ataxia (FRDA), an autosomal recessive disorder caused by mutations in Frataxin (FXN), a mitochondrial iron chaperone involved in iron-sulfur (Fe-S) clusters and heme biosynthesis [21,22,23,24]. Finally, reduced heme synthesis has been observed during aging [4,25].

In addition, other rare neurodegenerative disorders have been associated with defective heme transport across membranes. Several proteins are involved in this process [1,15]. Among them, Feline Leukemia Virus Subgroup C Receptor 1 (FLVCR1) and 2 (FLVCR2) are implicated in heme export and import, respectively [11,26,27,28,29,30,31]. Mutations in the heme exporter FLVCR1 are associated with three distinct disorders affecting the sensory nervous system: posterior column ataxia and retinitis pigmentosa (PCARP) [32,33,34], non-syndromic retinitis pigmentosa (RP) [35,36] and hereditary sensory and autonomic neuropathy (HSAN) [11,37]. Mutations in the heme importer *FLVCR2* are responsible for the Fowler syndrome, a proliferative glomerular vasculopathy [30,38,39].

Furthermore, several lines of evidence suggest that heme may also contribute to the pathogenesis of common neurodegenerative disorders. The deregulation of enzymes critically involved in heme synthesis has been reported in both Alzheimer’s disease (AD) and Parkinson’s disease (PD). Reduced 5-aminolevulinate synthase 1 (ALAS1) and porphobilinogen deaminase (PBGD) mRNA were observed in AD brains [40], suggesting decreased heme synthesis rates in AD. Moreover, heme deficiency has been reported in the brain of patients with AD [41]. It has been proposed that heme deficiency may arise from either decreased heme synthesis rates or heme depletion as a consequence of heme binding to amyloid-β [41,42,43]. However, increased Ferrochelatase (FECH) levels were reported in another study [41].

Heme binding to α-Synuclein has also been reported [44], suggesting that heme depletion may also occur in PD. In addition, blood transcriptomic meta-analysis showed downregulation of 5-aminolevulinate synthase 2 (ALAS2) and FECH in PD [45]. However, within PD erythroid cells, α-synuclein gene (SNCA) was co-expressed with crucial enzymes involved in heme metabolism, including ALAS2, FECH and biliverdin reductase B (BLVRB) [46]. Moreover, increased striatal 5-aminolevulinate dehydratase (ALAD) activity was observed in the MPTP-induced mouse model of PD, indicative of increased heme synthesis rates [47]. It is difficult to conclude from these studies whether heme synthesis is increased or reduced in AD and PD. Data are still controversial due to the low amount of human samples analyzed and the different experimental approaches adopted. Further studies are required to definitively determine heme synthesis rates in these pathological conditions. Mouse models of AD and PD will be extremely useful to analyze in detail the role of heme metabolism in these disorders.

A role for heme derived from extracellular sources in the pathogenesis of neurodegenerative disorders has also been proposed. The heme scavenger Hx has been found strongly increased in the cerebrospinal fluid of AD patients [48,49,50]. Similarly, altered expression of the hemoglobin scavenger haptoglobin was shown in AD [51,52], PD [53,54] and Huntington’s disease (HD) [55]. The induction of heme oxygenase 1 (HO1), the enzyme responsible for heme degradation, is common in patients affected by neurodegenerative conditions. Although HO1 is induced by a plethora of stimuli, it has been proposed that HO1 overexpression in AD and PD may be a consequence of increased brain-blood barrier permeability and hemoglobin-derived heme levels in AD and PD [16]. Moreover, increased expression of HO1 was reported in a mouse model of amyotrophic lateral sclerosis (ALS) [56] and ALS patients [57].

All the reported examples highlight the importance of the maintenance of heme homeostasis in the context of neurodegeneration. However, the molecular mechanisms underlying these disorders and the precise mechanism through which heme participates to them remains elusive and requires further investigations.

## 2. Role of Mitochondria in Neurodegenerative Diseases

**Figure 2:**
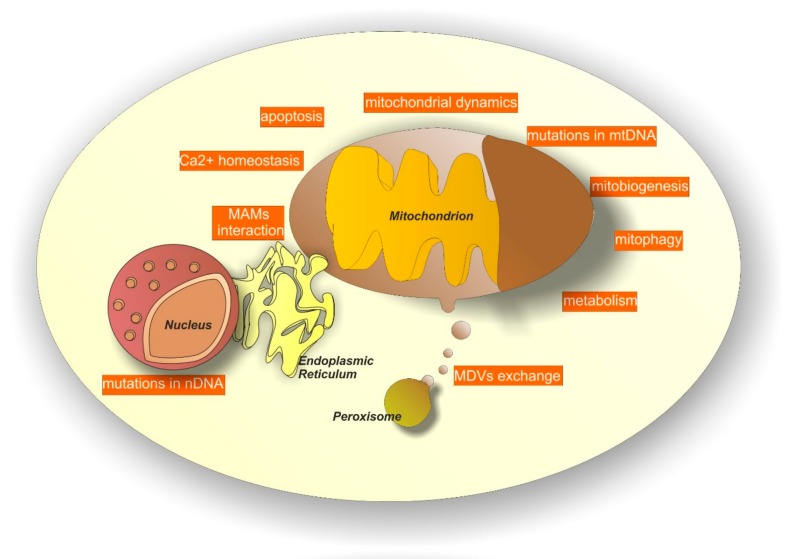
Mitochondrial dependent mechanisms in neurodegeneration. Mitochondria contribute to neurodegeneration by several mechanisms, including alterations in calcium homeostasis, mitochondrial biogenesis (mitobiogenesis), mitochondrial dynamics, metabolism and mitophagy. Moreover, mutations in mitochondrial DNA (mtDNA) and inappropriate activation of apoptosis can be alternative mechanisms. Finally, additional systems include mutations in nuclear DNA (nDNA) at the level of genes encoding for mitochondrial proteins, the compromised exchange of mitochondria-derived vesicles (MDVs) among mitochondria and peroxisomes and the inefficient interaction among mitochondria and the endoplasmic reticulum at the level of mitochondrial associated membranes (MAMs).

Neurodegeneration can be elicited by several systems. Among them, mitochondrial-dependent processes have become increasingly relevant [58,59,60,61,62,63]. Several mechanisms account for mitochondria-dependent neurodegeneration (see Figure 2 for a graphic list of them) and below we attempt to summarize the most important ones.

### 2.1. Mutations on Mitochondrial DNA (mtDNA) Genes

Mitochondria contain their own genome, which is made of multiple copies of a circular double stranded molecule, which is 16.6 kb long in humans. It comprises 37 genes, 13 encoding for proteins involved in adenosine triphosphate (ATP) production and the other 24 encoding for two rRNAs and 22 tRNAs. Cells contain thousands of molecules of mtDNA and the majority of them have the same sequence, a condition known as homoplasmy. Inefficient mtDNA repair, localized oxidative environment and increased replication, however, can promote mtDNA mutations that, due to the polyploidy nature of mtDNA, often co-exist with their wildtype counterpart in various proportions (a condition termed heteroplasmy). mtDNA mutations are usually responsible for defects in the respiratory chain functions, but only if they are present above a certain threshold level.

The replication of mtDNA occurs independently on cell cycle, and a particular mtDNA molecule may be strongly replicated (or not at all) during cell division. Moreover, replication occurs also in postmitotic cells. These phenomena account for the clonal expansion of mutated mtDNA molecules and in association with heteroplasmy, result in mosaicism, with the levels of mutated mtDNA varying dramatically between tissues in the same organism and in different regions of the same tissue.

Somatic mtDNA mutations accumulate during a person’s lifetime and undergo clonal expansion, so aging is typically associated with mosaic occurrence of respiratory chain-deficient cells in tissues [64].

Mitochondrial reactive oxygen species (ROS) production is the major cause for the higher mtDNA nucleotide instability when compared with nDNA.

Mitochondrial DNA mutations potently affect tissue that require a large amount of ATP to function, such as heart and brain. Some haplogroups [65] (evolutionary selected population subgroups carrying neutral single-base pair variants of mtDNA) have been associated with susceptibility to a variety of human diseases, including age-related neurodegenerative disorders such as PD and AD. Moreover, inherited point mutations and sporadic rearrangements on mtDNA have been described in association with neurodegeneration [65,66,67,68,69].

Although the presence of mtDNA deletions below a certain threshold is not sufficient to induce PD, small changes inside the genome of mitochondria could represent a risk factor for this pathology [70,71,72,73,74]. In addition, the accumulation of mutations in mtDNA over the course of PD has been observed to correlate with severity and burden of the disease [75]. Accumulation of mtDNA damage is also considered a possible mechanism of neurodegeneration over the course of HD [76]. Moreover, mtDNA mutations have been associated with AD [77], although the degree of mtDNA damage does not seem to correlate with the severity of AD symptoms [78].

As stated before, mtDNA alterations often result in defects on a particular component of the electron transport chain (ETC). The three main mechanisms through which mtDNA damage can contribute to neurodegeneration are therefore consequences of ETC alteration and include the decrease of ATP synthesis, the increase of ROS production and the enhanced sensitivity to neurotoxins associated to ETC disruption.

### 2.2. Mutations on Nuclear DNA Genes Encoding Proteins Crucial for Mitochondrial Functionality

Besides mutations on mitochondrial DNA, mutations in nuclear DNA (nDNA) at the level of genes encoding for mitochondrial proteins have also been associated with neurodegenerative disorders. For example, many of the identified ALS genes have a role in mitochondrial-associated functions (see [79] for a comprehensive description on this topic). In addition, mitochondrial dysfunction and oxidative stress in PD have been linked to mutations in genes encoding for parkin RBR E3 ubiquitin protein ligase (PRKN, commonly referred to as parkin), PTEN induced putative kinase 1 (PINK1, a protein that acts in the same pathway of parkin) and parkinsonism associated deglycase (DJ-1) [80], just to cite some of them. Parkin promotes autophagy of damaged mitochondria [81] and its deficiency is associated with defects in mitochondria morphology [82] and low levels of proteins involved in mitochondrial functions, thus resulting in decreased mitochondrial respiration [83]. Similarly, mutations in *PINK1* gene lead to decreased mitochondrial respiration [84] and alterations in mitochondria functions [85,86,87,88]. Regarding DJ-1, this protein localizes into mitochondria [89] and exerts crucial antioxidant functions [80,90,91,92]. Mutations in DJ-1 impair mitochondrial respiration, reduce mitochondrial membrane potential, increase ROS within the mitochondria and alter mitochondrial morphology [93]. Also, nDNA encoded proteins implicated in AD, like presenilin-1 and presenilin 2 (PSEN1 and 2) are related to mitochondria [94,95].

### 2.3. Alterations of Mitochondrial Dynamics (Fusion, Fission, Motility)

Mitochondria are not rigidly structured. They form a complex reticulum that undergoes regulated processes of fusion (the combination of two smaller mitochondria into a single organelle) and fission (the division of one large mitochondrion into two smaller fragments). Fusion allows mitochondria to mix their contents, enabling protein complementation, mtDNA repair and equal distribution of metabolites. Fission facilitates equal segregation of mitochondria into daughter cells during cellular division, enhances the distribution of mitochondria along cytoskeletal tracts and participates in the targeting of damaged segments of mitochondria to the autophagic process. Fusion and fission also contribute to the movement of mitochondria necessary for mitochondria distribution along neuronal axons and dendrites. Mitochondrial dynamics are crucial for neurotransmission, synaptic maintenance and neuronal survival. Proper mitochondrial trafficking is particularly important in neurons compared to other cell types, due to their exceptional cellular morphology. Indeed, neurons extend their axons and dendrites for very long distances that, in the case of human peripheral nerves or corticospinal tracts, extend up to a meter. Thus, the neuron represents an extreme case of mitochondrial distribution: dysfunctions in mitochondrial distribution that are not dangerous for other cells could be fatal for neuronal survival [96].

Alterations in mitochondria motility have been reported in several neurodegenerative disorders and neuropathies [97,98]. Aberrant activity of the fission-fusion machinery contributes to the pathogenesis of PD [99,100,101,102]. Moreover, alterations of mitochondrial dynamics have been observed in AD [61,102] and HD [100,103]. Particularly in HD, mitochondrial fission is promoted and mitochondrial fusion proteins are downregulated as the severity of the pathology increases [104]. Finally, defects in mitochondrial dynamics and disruption of the axonal transport of mitochondria have been reported in ALS [60,105,106,107].

### 2.4. Inappropriate Activation of Cell Apoptosis by Mitochondria

Mitochondria are pivotal organelles for the execution of apoptosis. The inappropriate activation of apoptosis leads to the disruption of the cellular proliferation-death balance. Neurodegenerative disorders are believed to partly depend on alterations of this equilibrium. *PINK1* loss-of-function mutations lead to early signals for apoptosis, promoting neurodegeneration in the context of PD [108]. Moreover, the low levels of *PTPA* (phosphotyrosyl phosphatase activator) observed in AD affected-people contribute to induce cell apoptosis in the brain of these patients [109]. Furthermore, caspase-6, an effector of the caspase-dependent apoptotic pathway, is known to be involved in the cleavage of mutant huntingtin resulting in neurodegeneration in HD patients [110]. Finally, in ALS the mutant SOD1 can trigger cytochrome c release from mitochondria to operate apoptosis [111].

### 2.5. Alteration of Mitochondria-Dependent Ca^2+^ Homeostasis

An additional neurodegenerative mechanism related to dysfunctions of mitochondria concerns the modulation of calcium. Mitochondria are involved in Ca^2+^ homeostasis as they are able to both accumulate and release Ca^2+^. Mitochondrial Ca^2+^ concentration is fundamental for the regulation of specific mitochondrial key functions, such as the apoptotic process and the activity of several mitochondrial enzymes. The deregulation of Ca^2+^ homeostasis is a hallmark of different neurodegenerative diseases including PD, AD, HD and ALS [112,113]. Moreover, alterations of calcium levels have been observed in neuropathies. Neuropathic pain phenotypes include chemotherapy induced neuropathy, diabetic neuropathy, human immunodeficiency virus (HIV)-associated neuropathy and Charcot-Marie-Tooth neuropathy. Neuropathies have been associated with mitochondrial dysfunctions [63], and particularly in diabetic neuropathy, impaired cellular calcium homeostasis, including alterations in mitochondrial Ca^2+^ concentration, has been reported. Indeed, in the context of diabetes, sensory neurons, above all the lumbar dorsal root ganglia neurons (which have the longest axons), show an increased intracellular Ca^2+^ concentration that triggers elevated mitochondrial Ca^2+^ levels. This condition induces mitochondrial membrane depolarization and can favor the generation of reactive oxygen species (ROS) and oxidative stress, as well as alterations in mitochondrial functionality that can ultimately lead to neuronal damage [114].

### 2.6. Additional Alterations of Mitochondrial-Related Processes: Biogenesis, Mitophagy, Mdvs Exchange, Interaction with Mams, Control of Cellular Metabolism

Besides the mechanisms reported above, a series of additional mechanisms involving mitochondria have been described. Among them, neurodegeneration has been associated with the impairment of mitochondrial biogenesis. Particularly, the deficit of peroxisome proliferator-activated receptor gamma coactivator-1α (PGC-1α), a key regulator of mitochondrial biogenesis, has been associated with HD, PD and AD [115,116,117,118,119,120].

Mitophagy is also linked to neurodegenerative disorders [121,122,123]. Mitophagy is the selective autophagic process responsible for the elimination of damaged or excess mitochondria. In this process, a peculiar role is played by PINK1, that recruits parkin to dysfunctional mitochondria, where it induces their degradation by mitophagy [124]. Thus, it is not surprising that in addition to other neurodegenerative disorders, defective mitophagy is highly implicated in PD and is considered one of the major pathological mechanisms of mitochondrial dysfunction in autosomal recessive forms of PD [125].

Mitophagy is a cellular process that eliminates whole mitochondria, but other mechanisms exist to partially eliminate portions or components of mitochondria [122]. Mitochondria-derived vesicles (MDVs) exchange between mitochondria and peroxisomes represent one of these possible mechanisms. MDVs are crucial for the transport of cargo from mitochondria to peroxisomes. This process is regulated by the retromer complex. Studies on vacuolar protein sorting 35 (VPS35), a component of the retromer complex, indicate that mutations or alterations in VPS35 expression are associated to PD [126,127]. Moreover, it has been demonstrated that Parkin and PINK1, two genes highly implicated in PD, play crucial roles in the control of this process [128,129].

Other than with peroxisomes, mitochondria also physically interact with other subcellular organelles to ensure efficient and rapid metabolism and signaling. For example, the interaction between mitochondria and the endoplasmic reticulum (ER) occurs at the level of MAMs (mitochondrial associated membranes), a subdomain of the ER. The proteins involved in neurodegenerative diseases such as DRP1 (dynamin related protein 1) and MFN2 (mitofusin 2) are enriched in MAMs [130,131] and the perturbation of mitochondria-ER contacts has been described in neurodegenerative disorders, including PD, AD and ALS [132].

A further mitochondrial-dependent mechanism has been highlighted in neurodegeneration. This is the impairment of cell metabolism [133]. Mitochondria are the main energy-producing organelles of the cell, thus any process impairing mitochondrial functionality may lead to metabolic switching aimed to compensate for their decreased ATP production. Among the different neurodegenerative disorders, many lines of evidence suggest that mitochondria-dependent mechanisms are responsible for the metabolic changes observed in dopaminergic neurons in the context of PD. Mitochondrial ROS are particularly abundant in dopaminergic neurons due to dopamine oxidative metabolism, enhanced Fenton’s reaction initiated by the high iron content of these cells, and the high rate of ATP production required to sustain the activity of a particular L-type voltage dependent Ca^2+^ channel expressed by these neurons. An excess of mitochondrial ROS can induce a series of cellular modifications, including hypoxia inducible factor 1α (HIF1α)-dependent up-regulation of glucose transporters [134], favoring the switch of energy metabolism towards glycolysis. When sustained for a long period, this metabolic change can be deleterious for dopaminergic neurons. Indeed, neurons need to deliver glucose in the pentose phosphate pathway (PPP), a process that produces NADPH (nicotinamide adenine dinucleotide phosphate), crucial for the recycling of the antioxidant glutathione. PPP is especially important in neurons as these cells show less robust antioxidant systems and are more vulnerable to oxidative stress than other cell types. Switching from PPP to glycolysis promotes oxidative stress and, consequently, neurodegeneration [135].

## 3. Heme and Mitochondrial Dysfunction Related to Neurodegenerative Diseases

**Figure 3:**
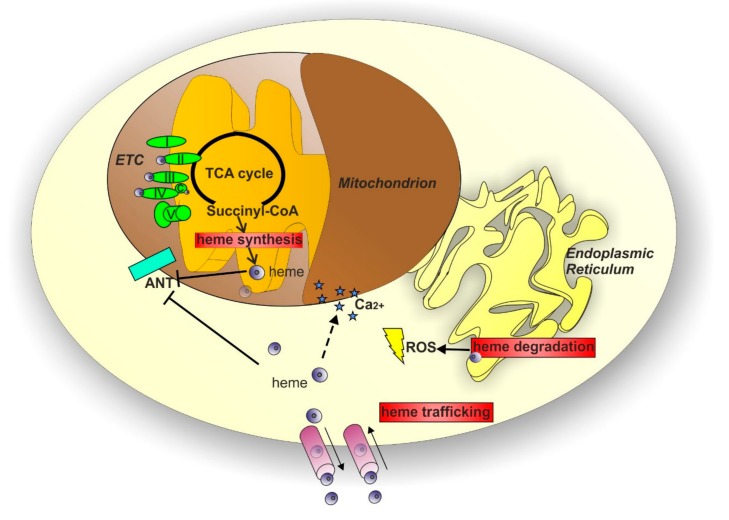
The “heme-mitochondria” relationship and the putative heme-related targets for the therapy of neurodegenerative disorders. Heme and mitochondria share a strong relationship based on several elements: heme synthesis occurs partly in the mitochondrion and acts as a cataplerotic pathway for the Kreb’s cycle; heme is a cofactor for cytochromes c and cytochromes in complexes II-III-IV of the mitochondrial ETC [137]; heme influences the ATP translocation between mitochondria and cytosol mediated by adenine nucleotide translocases (ANTs); heme export influences calcium (Ca^2+^) flux in mitochondria. Therefore, modulation of heme metabolism can lead to modification of mitochondrial functions. The control of intracellular heme levels is achieved by a balance among synthesis, catabolism and proper trafficking of heme. Thus, all these processes (highlighted with red boxes in the figure) represent putative good targets for the therapy of neurodegenerative disorder.

The examples reported above sustain the notion that mitochondrial dysfunctions play a critical role in neurodegenerative disorders. The mitochondrion is a critical organelle for cells, representing a crossroad for a plethora of reactions contributing to a variety of metabolic processes, including heme metabolism. Considering the impact of impaired heme homeostasis in several neurodegenerative disorders and the interesting potential that heme takes on for the research in the context of neurodegeneration, it is curious to notice that heme is an under-investigated molecule in the field. This discrepancy could be due to the lack of knowledge on the possible systems through which heme can influence pivotal processes implicated in neurodegeneration. Among the possible ways, it is tempting to speculate that heme could directly or indirectly affect many of the mitochondrial-dependent mechanisms of neurodegeneration described in the previous paragraphs. Indeed, the relationship between heme and mitochondria is based on several elements (Figure 3): heme is produced through a series of reactions that occur partly in the mitochondria and partly in the cytosol [136]; heme acts as a cofactor for cytochromes c and cytochromes in complexes II-III-IV of the mitochondrial ETC [137]; heme has been reported to directly or indirectly influence ATP translocation between mitochondria and cytosol [138,139,140] mediated by adenine nucleotide translocases (ANTs); finally, heme biosynthesis is considered a cataplerotic pathway for the Kreb’s cycle due to the fact that the first step of heme production consumes succynil-CoA [141,142]. Thus, modulation of heme homeostasis can affect mitochondrial functions.

Considering the mechanisms by which mitochondria contribute to neurodegeneration, it is interesting to note that heme levels can influence iron homeostasis, and both iron deficiency and iron excess are reported to cause damage on mitochondria [143,144] and on mitochondrial DNA [145,146]. Moreover, a decrease in heme itself leads to mitochondrial decay [4,147].

Furthermore, a connection exists between heme and nDNA genes encoding mitochondrial proteins typically implicated in neurodegenerative disorders. Indeed, it has been demonstrated that amyloid precursor protein, particularly when mutated, interacts and negatively regulates the heme-degrading enzyme HO1 [148]. Also PINK1 mutation is related to alterations in HO1 expression [149]. Moreover, DJ1 regulates nuclear factor-E2-related factor 2 (NRF2) [150], a key transcription factor for the induction of HO1 expression [151].

In addition, heme and the heme-degrading enzyme HO1 are implicated in the regulation of mitophagy, mitochondrial biogenesis and morphology [152,153,154].

In endothelial cells, it has been demonstrated that alterations in heme metabolism, in addition to promoting lipid peroxidation and activation of autophagy, induce mitophagy and apoptosis, indicating mitochondrial dysfunction [155]. Similarly, FLVCR1 loss is associated with alterations in mitochondrial morphology in human microvascular endothelial cells [26].

Furthermore, FLVCR1-deficient HeLa cells show impaired mitochondrial calcium uptake [156].

These data have been obtained in non-neuronal cells. However, similar mechanisms could also occur in neuronal cells.

Finally, compromised ETC complexes activity has been observed in the brain of a mouse model for acute intermittent porphyria, a type of porphyric neuropathy caused by alterations of heme biosynthesis [157]. Moreover, in three cases of Fowler syndrome it was suggested to be the presence of a defect in complex III and IV of the ETC [158,159].

These examples directly suggest that a connection between heme-mitochondria-neurodegeneration exists and open the possibility that future studies on this topic will further strengthen this notion.

## 4. Current Therapies and Potential Future Approaches to Face Mitochondrial Dysfunction in Neurodegenerative Diseases

Currently, there is no cure for reversing neurodegeneration and the treatment of neurodegenerative disorders is mainly symptomatic [160]. In order to face neurodegeneration, several pathways could be targeted to improve and/or restore mitochondrial functions, including mitochondrial biogenesis and metabolic flexibility, mitochondrial dynamics and mitophagy [161].

There are several pharmacological approaches to induce mitochondrial biogenesis and metabolic flexibility, that is the ability to switch from one fuel source to another. Several compounds have been generated to target the upstream sensors of energy production, including AMP-activated protein kinase (AMPK), mammalian target of rapamycin (mTOR) and sirtuins, or downstream transcriptional factors and co-factors, such as nuclear receptors, nuclear respiratory factor 1 (NRF1) and mitochondrial transcription factor A (TFAM) [161]. The therapeutic potential of these drugs has been evaluated mostly in the context of metabolic diseases, but also seems promising for neurodegeneration. For example, resveratrol is a natural compound that mimics caloric restriction and activates the sirtuin family of histone deacetylases. In humans, resveratrol improves mitochondrial function in obese patients and type 2 diabetes [162]. Furthermore, resveratrol counteracts neurodegeneration in worms and mice [163].

As described above, the disruption of the balance between mitochondrial fusion and fission contributes to neurodegeneration. Therefore, targeting mitochondrial dynamics represents another important strategy to improve mitochondrial function in neurodegenerative diseases. Strategies aimed at increasing mitochondrial fusion or inhibiting mitochondrial fission might improve mitochondrial function and are therapeutically interesting. The promotion of mitochondrial fusion by the overexpression of key components of the fusion machinery, like MFN2 or OPA1, rescues ATP production and mitochondrial morphology in a cellular model of PD [164]. Similar results were obtained with the inhibition of fission through the genetic deletion of DRP1 [164,165]. Although the understanding of the regulation of mitochondrial dynamics is still in its infancy, novel compounds have been identified to promote fusion or inhibit fission [166]. M1 hydrazone [167] and S3-derivative [168] promote mitochondrial fusion in cells deficient for mitofusin 1 (MFN1) and MFN2. Mdivi-1 (mitochondrial division inhibitor) attenuates fission in yeast and mammalian cells by inhibiting DRP1. In vitro, Mdivi-1 delays apoptosis by inhibiting mitochondrial outer membrane permeabilization and blocking cytochrome c release from mitochondria [169]. The therapeutic potential of Mdivi-1 seems promising for neurodegenerative disorders. Indeed, the administration of Mdivi-1 in mouse and cellular models of PD attenuates disease-associated phenotypes [165,170]. Although initially reported as an inhibitor of fission, Mdivi-1 was recently reported to reversibly inhibit complex I in a DRP1-independent manner [171]. The complete inhibition of complex I in vivo would be expected to cause neurodegeneration. Indeed, rotenone completely inhibits complex I, induces ROS levels and causes parkinsonian neurodegeneration in mice [172]. In contrast, Mdivi-1 lacks neuronal toxicity in vivo and is neuroprotective. This is likely due to the ability of Mdivi-1 to attenuate complex I-dependent reverse electron transfer (RET)-mediated ROS production. Indeed, Mdivi-1 fails to increase ROS levels in intact neurons and in isolated brain mitochondria [171].

The accumulation of dysfunctional mitochondria is another key event in several neurodegenerative conditions [173,174]. In this context, mitophagy is essential for the maintenance of mitochondrial integrity. As stated before in this review, the impairment of autophagy/mitophagy is common in neurodegenerative disorders. Therefore, mitophagy may be an additional pathway amenable for therapeutic intervention to ameliorate mitochondria function and counteract neurodegeneration [161,175]. Interestingly, both genetic and pharmacological induction of the mitochondrial autophagy receptor Nip3-like protein X (NIX) restores mitophagy in patient-derived fibroblasts [176].

Considering the crucial role of heme in maintaining mitochondrial function, it is tempting to speculate that targeting heme metabolism might be a promising strategy for the treatment of neurodegenerative diseases. Multiple approaches can be used to target heme metabolism (Figure 3); theoretically, targeting any of the enzymes involved in the heme biosynthetic pathway or proteins involved in the control of the intracellular heme pool may be a good strategy. Among these methods, HO1 represents a potentially interesting target. Due to its anti-oxidant and anti-inflammatory properties, HO1 plays a well-established neuroprotective role. The improvement of HO1 expression has been initially proposed for neurodegenerative conditions [177]. However, it has been reported that the overexpression of HO1 induces oxidative mitochondrial damage [178,179] and macroautophagy [179] in cultured astroglia. More importantly, HO1 induction has been associated with the later phases of neurodegeneration [180] and the deletion of HO1 has been proposed as a therapeutic option [181]. HO1 activity can be suppressed by synthetic metalloporphyrin compounds that unfortunately present important limitations [182]. However, novel HO1 inhibitors have been synthesized to overcome these side effects. Interestingly, these inhibitors confer neuroprotection in a mouse model of AD [182]. Considering the complex role of HO1 in neurodegeneration and the still controversial data reported in literature [180], further work is needed to fully elucidate the therapeutic potential of HO1 targeting. Recently, long-term 5–aminolevulinic acid (ALA) treatment has been exploited as a therapeutic approach in a mouse model of AD. Omori C. et al. reported that the oral administration of ALA increased cytochrome *c* oxidase (COX) activity and protein expression as well as mitochondrial membrane potential in the brain of treated mice [183]. Additional studies are required to understand more in detail the functional consequences of ALA administration in AD pathogenesis and the translation of this therapeutic approach to other neurodegenerative disorders. Considering that ALA formulations are already used for photodynamic therapy in a variety of cancer types [184], results obtained by Omori C. et al. are extremely encouraging for therapeutic purposes and further research in this direction is desirable.

## 5. Conclusions

The information reported over the course of the present review showed that mitochondria participate to neurodegenerative disorders by different mechanisms, encompassing DNA mutations, mitobiogenesis, mitophagy, mitochondrial dynamics, metabolism and mitochondrial interactions with other organelles. The literary contributions on the role of mitochondria in neurodegeneration are constantly growing and the present review attempted to make an excursus on the most important mechanisms by which these crucial organelles contribute to neurodegenerative diseases, with the awareness that not all the wide literature on this topic has been covered. Some of these mechanisms are currently considered as strategic targets for pharmacological interventions to counteract neurodegeneration. However, the investigation on additional elements contributing to the control of mitochondrial functions in neuronal cells will offer a wider window of intervention. In this perspective, heme metabolism provides an interesting opportunity. Indeed, as reported above in the review, heme participates to crucial processes occurring in mitochondria, influencing their functions and properties. The tight relationship between heme and the mitochondrion has been curiously underestimated and poorly investigated in the context of neurodegeneration. However, the implication of heme in crucial mitochondrial functions and the involvement of heme in a subset of neurodegenerative diseases strongly suggest an implication of heme in these disorders.

The comprehension of these mechanisms will allow the consideration of possible therapies based on the targeting of heme metabolism as an additional option to promote mitochondrial function. Alternatively, targeting heme metabolism may improve the efficacy of other drugs targeting mitochondria. The hope is that the understanding of the role of heme in mitochondria and its implication in neurodegeneration will open new perspectives in the struggle against neurodegenerative diseases.

## Figures and Tables

**Table 1:** Rare neurodegenerative disorders linked to defective heme metabolism.

*Disease*	*Gene*	*Inheritance*	*Clinical Features*	OMIM
ALAD deficiency	5-aminolevulinate dehydratase (ALAD)	autosomal recessive	Neuropathic Porphyria: acute neurovisceral attacks involving severe abdominal pain, peripheral neuropathies and psychiatric disturbances	612740
Acute intermittent porphyria (AIP)	Hydroxymethylbilane synthase (*HMBS*)	autosomal dominant		176000
Hereditary coproporphyria (HCP)	Coproporphyrinogen oxidase (*CPOX*)	autosomal dominant		121300
Variegate porphyria (VP)	Protoporphyrinogen oxidase (PPOX)	autosomal dominant		176200
Friederich Ataxia (FRDA)	Frataxin (FXN)	autosomal recessive	Progressive gait and limb ataxia associated with cardiomyopathy and diabetes	229300
Posterior Column Ataxia and Retinitis Pigmentosa (PCARP)	Feline Leukemia Virus Subgroup C Receptor 1 (FLVCR1)	autosomal recessive	Sensory ataxia and retinitis pigmentosa	609033
Non syndromic Retinitis pigmentosa (RP)	Feline Leukemia Virus Subgroup C Receptor 1 (FLVCR1)	autosomal recessive	Retinitis pigmentosa	268000
Hereditary Sensory and Autonomic Neuropathy (HSAN)	Feline Leukemia Virus Subgroup C Receptor 1 (FLVCR1)	autosomal recessive	Loss of pain perception	201300
Fowler syndrome (PVHH)	Feline Leukemia Virus Subgroup C Receptor 2 (FLVCR2)	autosomal recessive	Proliferative glomerular vasculopathy in the central nervous system associated with severe hydrocephaly, ventriculomegaly, cortical thinning and hypoplastic cerebellum.	225790
